# Occurrence patterns of crop‐foraging sika deer distribution in an agriculture–forest landscape revealed by nitrogen stable isotopes

**DOI:** 10.1002/ece3.8216

**Published:** 2021-10-11

**Authors:** Ayaka Hata, Rumiko Nakashita, Keita Fukasawa, Masato Minami, Yuko Fukue, Naoko Higuchi, Hikaru Uno, Yasuhiro Nakajima, Midori Saeki, Chinatsu Kozakai, Mayura B. Takada

**Affiliations:** ^1^ Institute of Livestock and Grassland Science National Agriculture and Food Research Organization (NARO) Tsukuba Ibaraki Japan; ^2^ Forestry and Forest Products Research Institute Tsukuba Ibaraki Japan; ^3^ Center for Environmental Biology and Ecosystem Studies National Institute for Environmental Studies Tsukuba Ibaraki Japan; ^4^ School of Veterinary Medicine Azabu University Sagamihara Kanagawa Japan; ^5^ Insutitute for Biodiversity Research and Education Earthworm Karuizawa Nagano Japan; ^6^ Advanced Analysis Center NARO Tsukuba Ibaraki Japan; ^7^ Faculty of Science and Engineering Chuo University Bunkyo‐ku Tokyo Japan

**Keywords:** agricultural crops, *Cervus nippon*, incidence function model, large ungulate, nitrogen stable isotopes, spatial distribution

## Abstract

Conflicts arising from the consumption of anthropogenic foods by wildlife are increasing worldwide. Conventional tools for evaluating the spatial distribution pattern of large terrestrial mammals that consume anthropogenic foods have various limitations, despite their importance in management to mitigate conflicts. In this study, we examined the spatial distribution pattern of crop‐foraging sika deer by performing nitrogen stable isotope analyses of bone collagen. We evaluated whether crop‐foraging deer lived closer to agricultural crop fields during the winter and spring, when crop production decreases. We found that female deer in proximity to agricultural crop fields during the winter and spring were more likely to be crop‐foraging individuals. Furthermore, the likelihood of crop consumption by females decreased by half as the distance to agricultural crop fields increased to 5–10 km. We did not detect a significant trend in the spatial distribution of crop‐foraging male deer. The findings of spatial distribution patterns of crop‐foraging female deer will be useful for the establishment of management areas, such as zonation, for efficient removal of them.

## Graphical abstract

We examined the spatial distribution pattern of crop‐foraging sika deer by performing nitrogen stable isotope analyses. Female deer in proximity to agricultural crop fields were more likely to be crop‐foraging individuals and the likelihood of crop consumption decreased by half as the distance to agricultural crop fields increased to 5–10 km.

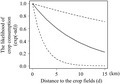

## 1 INTRODUCTION

With the expansion of human activities, reports of consumption of anthropogenic foods by wildlife are increasing worldwide (Oro et al., 2013). Access to anthropogenic foods by wildlife can increase human–wildlife conflict, resulting in economic losses (e.g., predation on agricultural crops and livestock) as well as increased risk of vehicle collisions and infectious disease transmission among wildlife, humans, and livestock (Becker et al., 2015; Cote et al., 2004; Honda et al., 2018; Johnson et al., 2020). Additionally, consumption of anthropogenic foods can affect wildlife population dynamics and local ecosystems via alterations in behavioral traits and physiological conditions (Gaynor et al., 2018; Hernando et al., 2020; Oro et al., 2013; Petroelje et al., 2019; Prange et al., 2004; Tucker et al., 2018). To mitigate these conflicts, it is important to develop management strategies for individuals that consume anthropogenic foods.

For managing anthropogenic food‐foraging wildlife, understanding their spatial distribution pattern is important to determine the efficient spatial allocation of management efforts. Large terrestrial animals in particular are known to move across long distances from their feeding sites, which may be located in artificial landscapes such as agricultural fields, to different habitats for shelter and digestion (Hata et al., 2017; Takada et al., 2002). Therefore, understanding the spatial distribution patterns of anthropogenic food‐foraging individuals is necessary to prioritize areas where management effort should be allocated to mitigate conflicts. Previous studies have investigated the spatial distribution of large terrestrial mammals by direct and indirect observations, such as radiotelemetry and camera trap surveys (Rubenstein & Hobson, 2004; Sanderson, 1966). However, these techniques have some limitations, including substantial effort and costs and inability to obtain information about the diet and geographical location of animals simultaneously (Hobson, 2005; Rubenstein & Hobson, 2004). Stable isotope analysis is an alternative tool that overcomes these weaknesses of conventional approaches (Crawford et al., 2008; Hobson et al., 2010). The stable isotope ratios in animal tissues are related to those in the diet (DeNiro & Epstein, 1978, 1981); when the isotope values differ between anthropogenic and natural food resources, animal tissues reflect the foraging history of anthropogenic foods (Demeny et al., 2019; Ditmer et al., 2016; Hata et al., 2017, 2021; Mizukami et al., 2005). It should be noted that stable isotope analysis needs to perform under the condition that animal tissues can be obtained in an ethically justifiable manner because this approach relies on animal tissue samples that usually collected from culled or immobilized individuals (except for hair samples collected by hair‐trap). While there are other approaches to obtain dietary information noninvasively such as DNA metabarcoding using scats (Shi et al., 2021; Thuo et al., 2019), stable isotope analysis has the advantage to provide insight into the long‐term dietary information of individual. However, only few studies have described the spatial distribution patterns of anthropogenic food‐foraging animals using stable isotope analysis (Hata et al., 2017; Walter, 2014).

Deer are typical large terrestrial mammals that move across multiple landscapes. In middle‐ to high‐latitude regions, deer often migrate seasonally in accordance with snow depth and food availability (Ball et al., 2001; Igota et al., 2004; Kufeld et al., 1989; Sabine et al., 2002). The consumption of anthropogenic foods, including agricultural crops, by deer has been documented and causes serious economic losses in many countries (Fagerstone & Clay, 1997; McCullough et al., 2009; Putman & Moore, 1998). Moreover, crop consumption has the potential to induce deer population growth (Hata et al., 2021; Iijima et al., 2013), which can increase agricultural crop damage and induce ecosystem changes; the increment of browsing pressure on the forest understory, the inhibition of woodland regeneration, and the promotion of fluctuations in the population and community structure of various taxa, from insects to mammals (Cote et al., 2004). To mitigate conflicts that arise from crop consumption by deer, it is necessary to understand spatial distribution patterns of crop‐foraging deer to manage them at an appropriate spatial scale.

In Japan, the consumption of crops, such as vegetables and pasture grasses, by sika deer (*Cervus nippon*) is well‐documented and causes serious economic losses (Ministry of Agriculture, Forestry & Fisheries, 2018). To mitigate conflicts, damage prevention management such as fencing and culling is conducted. Although many deer are killed every year (e.g., about 600,000 individuals were killed in Japan in 2019, in which about 23% were by hunting and 77% by culling) (Ministry of the Environment, 2020), agricultural damage by deer still amounts to over 50 million dollars every year (Ministry of Agriculture, Forestry, & Fisheries, 2018). More efficient and effective management strategies are needed to mitigate conflicts arising from crop consumption by deer. Sika deer inhabit various landscapes from plains to high‐altitude areas (Takatsuki, 2009) and migrate seasonally, as do most ungulates at middle and high latitudes (Igota et al., 2004; Takii et al., 2012; Takii Izumiyama, & Taguchi, 2012). Therefore, agricultural crop‐foraging deer may not consistently occur near agricultural crop fields throughout all seasons and may move long distances during the winter and spring, when crop production decreases. Because the food resources for deer are limited during the winter and spring (Seto et al., 2015; Yokoyama et al., 2000), deer culling is suitable during these seasons when the bait‐trap success improves, and shooting also can be easy with better visibility without leaves. Therefore, clarifying the spatial distribution pattern of crop‐foraging deer during these seasons will facilitate the efficient spatial allocation of management efforts to mitigate conflicts.

In this study, we examined the spatial distribution pattern of crop‐foraging sika deer during the winter and spring in central Japan. We investigated crop consumption by performing nitrogen stable isotope analyses of bone collagen samples. The nitrogen stable isotope ratios (δ^15^N) of bone collagen were expected to reflect the foraging history and crop consumption by individual deer (Hata et al., 2021). We examined whether deer likely to consume crops live closer to agricultural crop fields, even during the winter and spring.

## 2 MATERIALS AND METHODS

### 2.1 Study area

**FIGURE 1:**
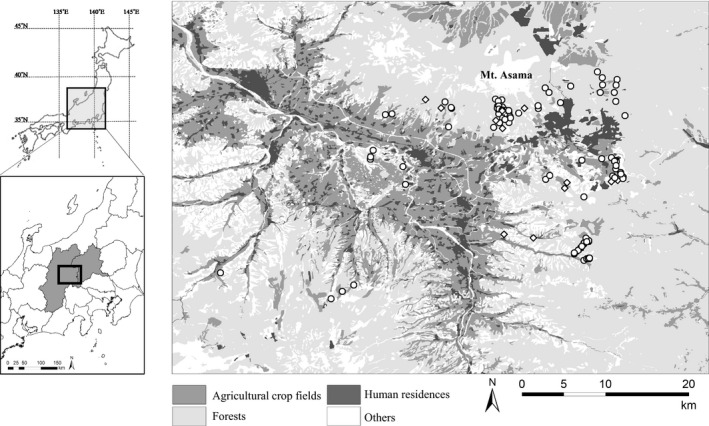
Location of the study area in eastern Nagano and western Gunma Prefectures in central Japan. In this area, we collected skull specimens of sika deer (*Cervus nippon*) that were hunted by hunters or in animal control kills. Between late December and early May, female and male deer were killed from 2012 to 2020 and in 2020, respectively. We also collected data for location of the deer (latitude and longitude) for each individual. Female and male deer specimens were collected by shooting and trapping and by trapping only, respectively. Each point indicates the location where a female (circle) or male (rhombus) deer was killed

We studied the sika deer population inhabiting eastern Nagano and western Gunma Prefectures in central Japan (Figure 1). The landscape of the study area is an agriculture–forest mosaic (Figure 1), including broad‐leaved trees, such as *Juglans* sp., *Quercus crispula*, and *Cornus controversa*, and coniferous trees, including *Cryptomeria japonica* and *Chamaecyparis obtusa*. This area also has mountainous areas with broad‐leaved trees, such as *Q*. *crispula* and *Betula platyphylla*, coniferous trees, including *Larix kaempferi* and *Abies mariesii*, and an alpine zone (Institute for Biodiversity Research & Education Earthworm, 2014). Agricultural crop fields comprised both crop fields and sown grasslands because deer consume vegetables and pasture grasses in this area (Hata et al., 2019; Nagano Prefecture, 2016; Tsukada et al., 2012). To our knowledge, we considered there are no other food items except for agricultural crops that may arise the δ^15^N values of deer in this study area. The elevation of the study area ranged from about 700 to 2500 m. The average of maximum snow depth at the foothill of Mt. Asama was 38 cm in 2012–2019 (Japan Meteorological Agency, 2020). The estimated deer density in this area was 31 individual/km^2^ in 2015 (Nagano Prefecture, 2016).

### 2.2 Data collection

In the study area, 147 skull specimens of deer hunted by local hunters or in animal control culls were collected (Table S1). Females were killed from 2012 to 2020, and males were killed in 2020. All specimens of deer were killed between late December and early May in each year. Data for location of the deer (latitude and longitude) were collected for each individual. For female deer (*n* = 128), 38 and 90 specimens were collected by shooting and trapping, respectively. For male deer (*n* = 19), all specimens were trapped. Deer culling by shooting was conducted only at high elevations (1000–1800 m) in mountainous areas.

### 2.3 Stable isotope analysis

To estimate the relative dietary contribution of crops in deer, nitrogen stable isotope analysis of bone collagen was performed. Bone collagen has a relatively slow turnover rate and therefore provides dietary information spanning several years or the lifetime of the individual (Hedges et al., 2007; Koch, 2007; Stenhouse & Baxter, 1979). The δ^15^N values of crops such as vegetables and pasture grasses are much higher than those of wild plants (Hata et al., 2021). Thus, the δ^15^N values of bone collagen reflect the long‐term history of the relative dietary contribution of crops in deer (Hata et al., 2021). Bone collagen was obtained by collagen extraction from bone fragments of the nasal turbinate obtained from the skull specimen. Collagen was extracted following the methods described by Hata et al. (2021). The samples were enclosed in a tin cup and combusted in an elemental analyzer (FlashEA1112; Thermo Fisher Scientific) interfaced with an isotope ratio mass spectrometer (Thermo Scientific Delta V Advantage; Thermo Fisher Scientific), which was used to analyze the nitrogen isotope ratios. Nitrogen isotope ratios are expressed in delta (δ) notation as parts per thousand (‰) relative to *R*_standard_ as follows:

$$\delta \left(‱\right)=\left[\left(R_{\text{sample}}/R_{\text{standard}}\right)‐1\right]\times 1000.$$

where *R*_sample_ and *R*_standard_ are ^15^N/^14^N ratios of the samples and the standard, respectively. The standard is the isotope ratio of atmospheric nitrogen (AIR). The analytical error for the isotope analysis was within 0.1‰ for δ^15^N.

### 2.4 Statistical analysis

We assessed the effect of the accessibility to agricultural crop fields from the location of deer killed on the δ^15^N values of bone collagens (i.e., the relative dietary contribution of crops). Because the lactation can affect the δ^15^N values (Tsutaya & Yoneda, 2015) and that effect may be particularly large in 0‐year‐old deer, we tested for differences in the δ^15^N values of bone collagens between 0‐ and ≥1‐year‐old by two‐sample *t* test. Because there were no differences between them (*t* = 0.13, df = 126, *p* = .90), we pooled all ages and used for subsequent analyses. We aggregated the area (m^2^) of agricultural crop fields by the “1 × 1 km mesh (the third mesh),” a national grid system of Japan with a unit cell size of 30″ in latitude and 45″ in longitude (c. 1 × 1 km) using GIS software (ArcGIS Desktop 10.4.1). The classification of landscape elements in the study area was based on the most recent vegetation/land‐use map available on J‐IBIS (Ministry of the Environment, 1999). As mentioned above, we classified both crop fields and sown grasslands as agricultural crop fields because deer consume vegetables and pasture grasses in the area (Hata et al., 2019; Nagano Prefecture, 2016; Tsukada et al., 2012).

Accessibility to agricultural crop fields was expressed using an accessibility index defined by the incidence function model (IFM, Hanski, 1994). The IFM is a useful measure of connectivity that incorporates both areas of potentially accessible patches (i.e., crop field in 1 × 1 km grid cells) and the distance to these patches. We considered that the IFM would be more suitable than simpler indices such as the distance to nearest agricultural crop fields and per cent agricultural crop fields within a buffer radius because the IFM is based on a realistic assumption that deer individuals can access agricultural crop fields at multiple grid cells within their home ranges and incorporates smooth distance decay in accessibility. Because the migratory behavior and dispersal pattern can vary according to sex (Takii, Izumiyama, & Taguchi, 2012), separate models were generated for males and females. As data obtained by field observations often contain spatially correlated errors which can decrease effective sample sizes, we also explicitly modeled the correlation structure of error term in space.

The following model was constructed to evaluate the effect of the accessibility to crop fields on the δ^15^N values in deer:

$$y_{i}=\mu_{i}+e_{i}.$$

$$\mu_{i}=\beta_{0}+\beta_{1}\Sigma_{j}\exp\left(‐\alpha d_{\mathrm{ij}}\right)A_{j}+\beta_{\text{2Ci}}.$$

where *y_i_* is the *i*th δ^15^N value of bone collagen, reflecting the feeding history over several years or the life span of the individual (Hedges et al., 2007; Koch, 2007; Stenhouse & Baxter, 1979). This parameter was used as the relative dietary contribution of crop of each deer individual. The accessibility index was defined by Σ*_j_* exp(−*αd_ij_*)*A_j_*, where α is a parameter controlling for mobilization ability with respect to distance (i.e., small α meaning slow distance decay). It is a special case of the original IFM (Hanski, 1994), $\Sigma_{j}\exp\left(‐\alpha d_{\mathrm{ij}}\right)A_{j}^{\beta }$, when *β* = 1, assuming linearity between the area of the crop field and the amount of accessible resource (i.e., agricultural crop) in the field. The straight‐line distance between the *i*th location of deer killed and mesh *j* was denoted by *d_ij_*, and the crop field area of the *j*th grid cell was denoted by *A_j_*. Because agricultural crops are often used for baits (Ikeda et al., 2018; Kilpatrick et al., 2010), crop‐foraging deer may be more familiar with trapping baits and more likely to consume them; that is, the δ^15^N values may be higher for deer killed by trapping than by shooting. Therefore, we added the method of culling (*C*) as a confounding factor (set to 1 for shooting and 0 for trapping). *β*_0,_ *β*_1_, and *β*_2_ are the intercept, the coefficient for Σ*_j_* exp(−*αd_ij_*)*A_j_*, and the coefficient for *C*, respectively. Vector *e* = (*e*_1_, *e*_2_, … , *e*_N_) is a spatially correlated error term following a multivariate normal distribution with mean vector 0 and variance–covariance matrix Σ. The Σ contains both spatially structured and unstructured (i.e., white noise) part as follows:

$$\Sigma =\sigma_{1}^{2}C+\sigma_{2}^{2}I$$

where *σ*_1_ and *σ*_2_ are parameters controlling magnitudes of spatially structured and unstructured errors, respectively. The I is an identity matrix, and C is a correlation matrix between sample locations, *i*_1_ and *i*_2_. An element of C, $C_{i_{1}i_{2}}$, was modeled so that correlation between locations decays exponentially:

$$C_{i_{1}i_{2}}=\exp\left(‐D_{i_{1}i_{2}}/k\right)$$

where $D_{i_{1}i_{2}}$ is a distance between *i*_1_ and *i*_2_, and *k* is a parameter controlling the range of autocorrelation. By definition, the diagonal element of C, *C_ii_*, is always 1. The values of ln(α), *β*_0_, *β*_1_, *β*_2_, ln(*σ*_1_), ln(*σ*_2_), and ln(*k*) were estimated using the maximum likelihood method. Log likelihood was calculated by multivariate normal distribution as follows,

$$L\left(\ln\left(\alpha \right),\;\beta_{0},\beta_{1}{,\beta }_{2},\;\ln\left(\sigma_{1}\right),\ln\left(\sigma_{2}\right),\ln\left(k\right)\right)=‐\frac{N}{2}\ln\left(2\pi \right)‐\frac{1}{2}\ln\left(\left|\Sigma \right|\right)‐\frac{1}{2}{\left(y‐\mu \right)}^{T}\Sigma^{‐1}\left(y‐\mu \right)$$

where *y* = (*y*_1_, *y*_2_, … , *y_N_*) is the vector of response variable, *μ* = (*μ*_1_, *μ*_2_, …, *μ_N_*) is the mean vector, and ∑ is the covariance matrix defined above. We searched maximum likelihood estimate of parameter vector, which maximizes log likelihood function using R function optim (). In male model, the *β*_2_ *C* term was absent because all male deer were killed by trapping only. Spatial autocorrelation was not considered in male model because the model was not converged. All statistical analyses were performed using R for Windows 3.5.2 (R Development Core Team, 2018).

## 3 RESULTS

The average δ^15^N values for female and male deer were 3.1‰ (range −1.1‰ to 7.3‰) and 2.4‰ (range 0.5‰–4.1‰), respectively (Table S1). The average δ^15^N values for female deer killed by trapping and shooting were 3.3‰ (range −0.5‰ to 7.3‰) and 2.7‰ (range −1.1 to 5.2‰), respectively (Table S1).

**FIGURE 2:**
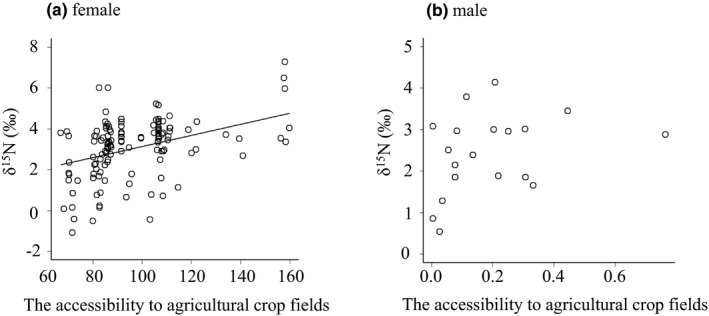
Relationship between the accessibility to agricultural crop fields and the δ^15^N values for (a) female and (b) male deer. Accessibility ∑*_j_* exp(−*αd_ij_*)A*_j_* was expressed using an accessibility index defined by the incidence function model (Hanski, 1994). Accessibility on the *x*‐axis was calculated based on the maximum likelihood of ln(*α*), and therefore, the scale of accessibility for females and males was different. A regression line is only shown for females because the accessibility to agricultural crop fields was positively related to the δ^15^N values only in the female model (Table 1)

**FIGURE 3:**
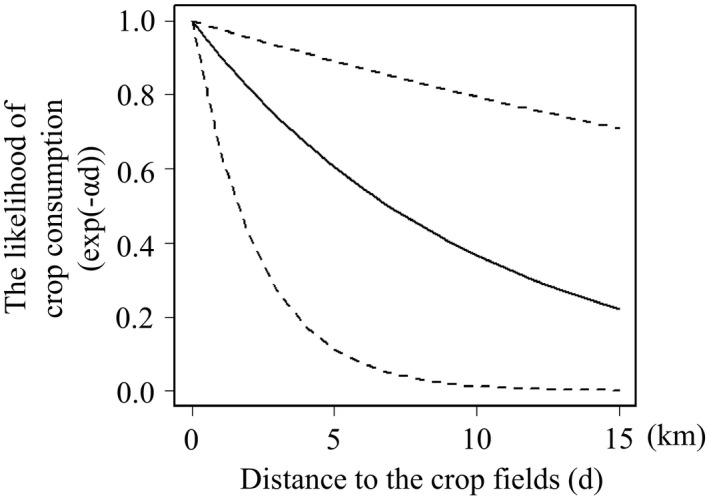
Relationship between the distance from the location of deer killed to agricultural crop fields (*d*) and the likelihood of crop consumption (exp(−*αd*)) for females. The likelihood of crop consumption completely depended on the agricultural crop field area in each 1 × 1 km mesh where *d* is zero and decreases rapidly with increasing *d* (see Section 3). The solid line shows the mean, and dashed lines indicate the 95% CI

In the female model, accessibility to agricultural crop fields was positively related to the δ^15^N values (Table 1, Figure 2). The log‐transformed scaling factor ln(*α*) was 2.30 (Table 1). The α determines the distance dependence in accessibility to a crop field illustrated by exp (−*αd*), which provides a measure of the relative crop consumption of female deer in relation to the distance to agricultural crop fields (*d*; Figure 3). Figure 3 shows that where *d* is zero, the likelihood of crop consumption completely depends on the crop field area in each 1 × 1 km mesh. The likelihood of crop consumption decreased by half when the distance to agricultural crop fields (*d*) was 5–10 km (Figure 3). The method of culling did not affect the δ^15^N values (Table 1). In the model for male deer, accessibility to agricultural crop fields did not affect the δ^15^N values (Table 1; Figure 2). ln(*α*) was −0.66 (Table 1).

**TABLE 1:** Estimates of parameters for female and male models

Parameters	Female					Male				
	Estimates	SE	95% CI	*z* value	*p* Value	Estimates	SE	95% CI	*z* value	*p* Value
ln(*α*)	2.30	0.75	0.83–3.77	—	—	−0.66	1.16	−2.94 to 1.62	—	—
*β_0_*	0.44	1.85	−3.17 to 4.06	—	—	2.11	0.34	1.44–2.78	—	—
*β*_1_	0.55	0.13	0.29–0.80	4.19	<.001	0.31	0.21	−0.10 to 0.72	1.49	.14
*β*_2_	−0.37	0.32	−1.00 to 0.26	−1.16	.25	—	—	—	—	—
ln(*σ*_1_)	0.14	0.10	−0.07 to 0.34	—	—	—	—	—	—	—
ln(*σ*_2_)	−0.40	0.12	−0.63 to −0.16	—	—	−0.12	0.16	−0.44 to 0.20	—	—
ln(*k*)	−2.80	1.35	−5.44	—	—	—	—	—	—	—

Note

In male model, the *β*_2_ *C* term was absent because male deer were killed by trapping only. Spatial autocorrelation was not considered in male model because the model was not converged.

## 4 DISCUSSION

Our isotopic approach revealed the spatial distribution pattern of crop‐foraging female sika deer. Female deer in proximity to agricultural crop fields during the winter and spring were more likely to be crop‐foraging individuals (Figure 2). Possible reasons why crop‐foraging deer distributed in proximity to agricultural crop fields are as follows. Although seasonal migration is well known in cervids inhabiting middle‐ to high‐latitude regions (Igota et al., 2004; Monteith et al., 2011; Mysterud, 1999; Takii, Izumiyama, Mochizuki, et al., 2012), sedentary behavior has also been observed in accordance with food availability (Igota et al., 2004; Kufeld et al., 1989). In general, the availability of both crops and natural food resources is low during the winter and early spring. However, crops do not completely disappear. In fact, deer aggregate in sown grasslands to consume pasture grasses under snow during the snowy season (Hata et al., 2019), and many leftover vegetables remain in agricultural crop fields for long periods (A. Hata, personal observation). Therefore, deer that are more likely to consume crops might tend to distribute in proximity to agricultural crop fields for crop consumption, even during the winter and spring. In fact, it was reported that two female sika deer tracked with Global Positioning System (GPS) inhabited areas near agricultural crop fields throughout the year (Ishizuka et al., 2007).

The likelihood of crop consumption by females decreased by half as the distance to agricultural crop fields increased to 5–10 km (Figure 3). Accordingly, crop‐foraging female deer are more likely to distribute within this range. The width of the range may depend on behavioral variation among deer, such as migration. To elucidate the determinants of behavioral differences among crop‐foraging individuals, further research is needed with consideration of additional factors, such as the response to hunting risk (Kamei et al., 2010; Kilgo et al., 1998; Little et al., 2016; Lone et al., 2015), genetic inheritance (Mueller et al., 2013), and social learning (Hopkins, 2013). Nevertheless, our findings can be useful to allocate management efforts such as culling at the appropriate spatial scale to mitigate conflicts that arise from crop consumption by deer.

In contrast to our results for female deer, there was no significant trend in the spatial distribution pattern of crop‐foraging male deer (Figure 2). As with many mammals (Pusey, 1987), male deer show a greater tendency to disperse from their natal area than females. In fact, dispersal movement has been observed in juvenile male sika deer (Takii, Izumiyama, & Taguchi, 2012; Yamazaki & Furubayashi, 1995). Takii, Izumiyama, and Taguchi (2012) reported that some males dispersed at 1–2 years old with distances of 3.0–40.3 km. In our data, eight males were 1–2 years old (Table S1). Even if they inhabited nearby agricultural crop fields with their mothers and consumed agricultural crops when they were young, they might disperse distant other habitats. The differences between sexes in movement behaviors might affect spatial distribution of crop‐foraging deer. The small sample size of males might have decreased the statistical power of the analyses. Further studies are needed to understand the characteristics of movement of crop‐foraging males.

Our results for the spatial distribution patterns of crop‐foraging female deer provide a basis for management at an appropriate spatial scale to mitigate conflicts. Because crop‐foraging female deer were more likely to distribute within 5–10 km of agricultural crop fields, the establishment of management areas, including zonation, based on this result might be effective. Intensive culling in this zone would allow for effective removal of crop‐foraging female deer. Culling crop‐foraging female deer is expected to suppress deer population growth because crop consumption induces precocious maturity and may promote population growth (Hata et al., 2021). Moreover, intensive culling using zonation can prompt deer to flee from nearby agricultural crop fields because deer avoid hunting activity by altering their behavior and habitat utilization (Kamei et al., 2010; Kilgo et al., 1998; Little et al., 2016; Lone et al., 2015). In the future, it is necessary to evaluate the effectiveness of intensive culling in the management areas to decrease crop‐foraging deer and subsequent crop damage and ecological impacts.

## CONFLICT OF INTEREST

The authors declare that they have no conflict of interest.

## AUTHOR CONTRIBUTIONS

**Ayaka Hata:** Conceptualization (lead); Data curation (lead); Formal analysis (equal); Funding acquisition (lead); Investigation (equal); Writing‐original draft (lead); Writing‐review & editing (equal). **Rumiko Nakashita:** Conceptualization (supporting); Data curation (equal); Investigation (equal); Resources (equal); Writing‐review & editing (supporting). **Keita Fukasawa:** Conceptualization (supporting); Formal analysis (equal); Methodology (lead); Software (lead); Writing‐review & editing (equal). **Masato Minami:** Data curation (equal); Investigation (equal); Resources (equal). **Yuko Fukue:** Data curation (equal); Investigation (equal); Resources (equal). **Naoko Higuchi:** Data curation (equal); Investigation (equal); Resources (equal). **Hikaru Uno:** Investigation (equal); Resources (equal). **Yasuhiro Nakajima:** Investigation (supporting); Resources (equal). **Midori Saeki:** Data curation (equal); Investigation (equal); Writing‐review & editing (supporting). **Chinatsu Kozakai:** Conceptualization (supporting); Formal analysis (equal); Writing‐review & editing (supporting). **Mayura Takada:** Conceptualization (equal); Formal analysis (equal); Supervision (lead); Writing‐review & editing (equal).

## Supporting information

Table S1

## Data Availability

The sample data used in this study are available in the supporting information (excel file). The R script and related data are available on Dryad (https://doi.org/10.5061/dryad.jwstqjq9g).
